# Utility of new FDG-PET/CT guidelines for diagnosing cardiac sarcoidosis in patients with implanted cardiac pacemakers for atrioventricular block

**DOI:** 10.1038/s41598-024-58475-z

**Published:** 2024-04-03

**Authors:** Subaru Tanabe, Yusuke Nakano, Hirohiko Ando, Masanobu Fujimoto, Tomohiro Onishi, Hirofumi Ohashi, Shimpei Kuno, Kazuhiro Naito, Katsuhisa Waseda, Hiroshi Takahashi, Yasushi Suzuki, Motoyuki Fukuta, Tetsuya Amano

**Affiliations:** 1 Department of Cardiology, Aichi Medical University, 1-1 Yazakokarimata, Nagakute, Aichi 480-1195 Japan; 2 Fujita Health University School of Medical Science, 1-98 Dengakukubo, Kutsukake, Toyoake, Aichi Japan; 3 Department of Cardiology, Tajimi City Hospital, 3-43 Maehatacho, Tajimi, Gifu Japan

**Keywords:** Cardiology, Diseases, Signs and symptoms

## Abstract

Diagnosing cardiac sarcoidosis (CS), especially in isolated cases, is challenging, particularly due to the limitations of endomyocardial biopsy, leading to potential undiagnosed cases in pacemaker-implanted patients. This study aims to provide real world findings to support new guideline for CS using 18F-fluoro-deoxyglucose positron-emission tomography computed tomography (FDG-PET/CT) which give a definite diagnosis of isolated CS (iCS) without histological findings. We examined consecutive patients with cardiac pacemakers for atrioventricular block (AV-b) attending our outpatient pacemaker clinic. The patients underwent periodical follow-up echocardiography and were divided into two groups according to echocardiographic findings: those with suspected CS and those without suspected CS. Patients suspected of having nonischemic cardiomyopathy underwent FDG-PET/CT for CS diagnosis. We investigated the utility of the new guideline for CS using FDG-PET/CT. Among the 272 patients enrolled, 97 patients were implanted with cardiac pacemakers for AV-b. Twenty-two patients were suspected of having CS during a median observation period of 5.4 years after pacemaker implantation. Of these, one did not consent, and nine of 21 cases (43%) were diagnosed with definite CS according to the new guidelines. Five of these nine patients were diagnosed with iCS using FDG-PET/CT. The number of patients diagnosed with definite CS using the new guidelines tended to be approximately 2.3 times that of the conventional criteria (p = 0.074). Three of the nine patients underwent steroid treatment. The composite outcome, comprising all-cause death, heart failure hospitalization, and a substantial reduction in left ventricular ejection fraction, were significantly lower in patients receiving steroid treatment compared to those without steroid treatment (p = 0.048). The utilization of FDG-PET/CT in accordance with the new guidelines facilitates the diagnosis of CS, including iCS, resulting in approximately 2.3 times as many diagnoses of CS compared to the conventional criteria. This guideline has the potential to support the early identification of iCS and may contribute to enhancing patient clinical outcomes.

## Introduction

Sarcoidosis is a systemic inflammatory disease associated with the formation of noncaseating granulomas and subsequent tissue scarring of unknown etiology^1^. Initiating immunosuppressant therapy from the early active phase is suggested for the treatment of patients with clinical manifestations of cardiac sarcoidosis (CS), as patients with CS who develop cardiac dysfunction due to high scar burden have poorer prognoses^2–4^. However, as conduction abnormalities such as atrioventricular block (AV-b) can be the first and sometimes only manifestation of CS, it is difficult to make an early definite diagnosis of CS, especially in patients with isolated CS (iCS) without any specific findings in other organs^1,5,6^. This challenge is exacerbated by the low diagnostic accuracy of endomyocardial biopsy, which is currently the only technique for the pathological diagnosis of CS^7^. Therefore, patients implanted with cardiac pacemakers may have undiagnosed CS and develop cardiac dysfunction after cardiac pacemaker implantation^1^.

The 2016 update to the guidelines by the Japanese Circulation Society (JCS) regarding the diagnosis and treatment of CS changed the diagnostic criteria and established the initial definition of iCS (Supplementary Table 1)^8^. Moreover, in the new guideline, iCS can be diagnosed without histological findings^8,9^, even though international guidelines, including the conventional JCS 2006 guidelines for diagnosing CS, require histological evidence of noncaseating granulomas in at least one organ^10–12^. The major criteria for the diagnosis of CS were expanded to include the use of 18F-fluoro-deoxyglucose positron-emission tomography computed tomography (FDG-PET/CT) and gadolinium-enhanced magnetic resonance imaging (MRI), allowing for the detection of inflammatory lesions in the myocardium of patients with suspected CS^8,13^.

This new guideline may provide a definite diagnosis of undiagnosed CS and clinical benefits for prognosis; however, real-world evidence is limited. Thus, the purpose of this study was to investigate the utility of the new guideline utilizing FDG-PET/CT to diagnose CS and its clinical implication among patients implanted with cardiac pacemakers for AV-b.

## Results

### Study populations and baseline clinical characteristics

**Figure 1:**
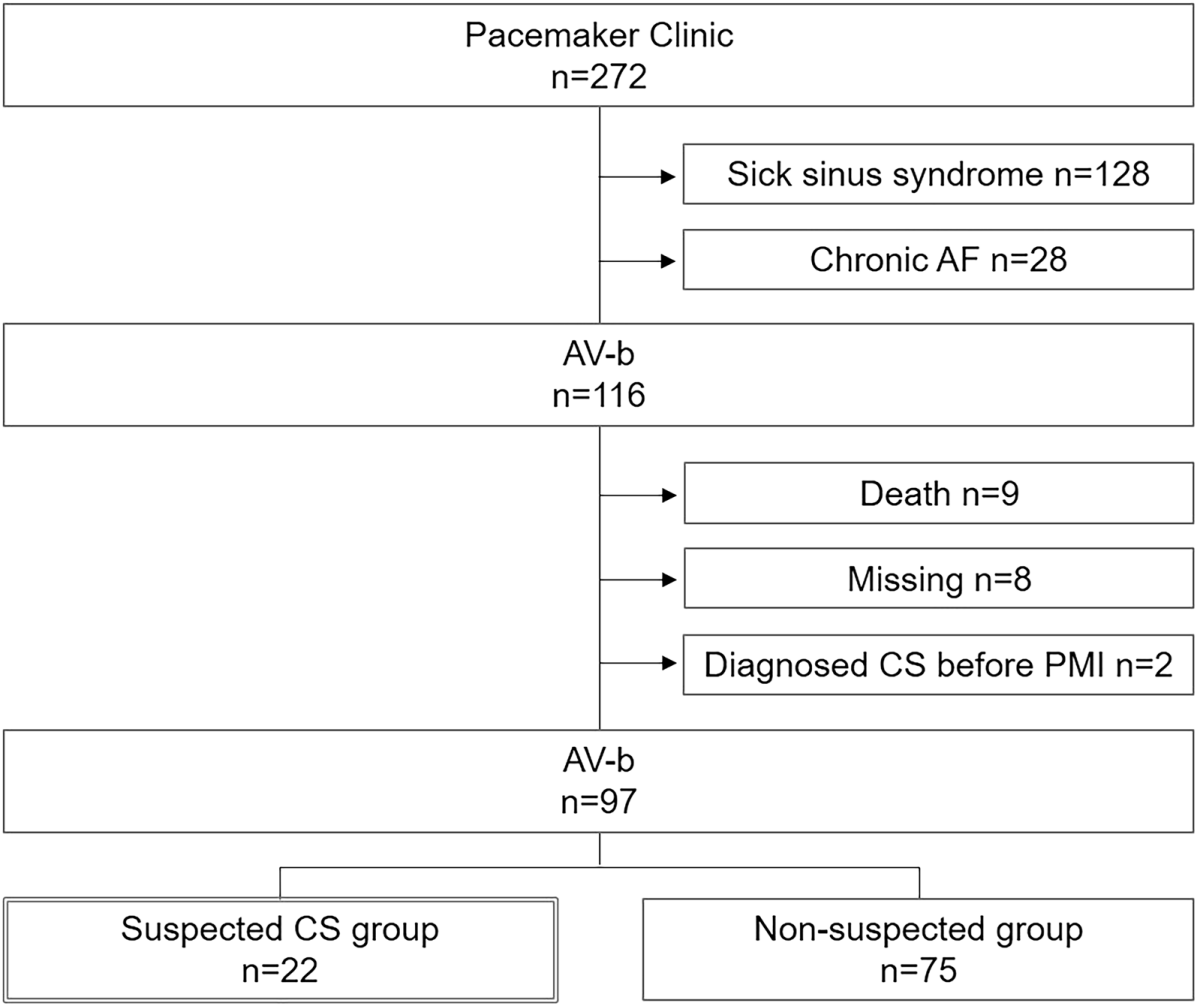
Flow chart of patient enrollment. Among 272 consecutive patients with a cardiac pacemaker who attended a pacemaker clinic, 116 who had received cardiac pacemaker implantation for atrioventricular block were recruited. Patients who died (n = 9) or were missing (n = 8) during the follow-up period and those who had already been diagnosed with CS (n = 2) were excluded. Finally, 97 patients were divided into two groups at a median follow-up of 5.4 years after cardiac pacemaker implantation: 22 (22.7%) to the suspected CS group (LVEF ≤ 50% or ventricular structural abnormality) and 75 (77.3%) to the non-suspected CS group (LVEF > 50% without any abnormality). *AF* atrial fibrillation, *AV-b* atrioventricular block, *CS* cardiac sarcoidosis, *LVEF* left ventricular ejection fraction, *PMI* pacemaker implantation.

In total, 272 consecutive patients with cardiac pacemakers in our pacemaker clinic were enrolled in this study. Patients who underwent cardiac pacemaker implantation for sick sinus syndrome (n = 128) or chronic AF (n = 28) were excluded. Moreover, patients who died (n = 9) or were missing (n = 8) during the follow-up period were excluded. Missing data included patients who were transferred to other hospitals and those for whom examinations were difficult. Furthermore, patients who had already been treated for CS were excluded (n = 2). Finally, 97 patients were divided into two groups at a median follow-up of 5.4 years after cardiac pacemaker implantation. The suspected CS group included 22 patients (22.7%; 22/97) and the non-suspected CS group included 75 patients (77.3%; 75/97) (Fig. 1).

The baseline clinical characteristics of the patients at the time of group assignment are shown in Table 1. The median age was significantly younger in the suspected than in the non-suspected CS group (71.0 vs. 81.0 years, p = 0.002), and the proportion of males tended to be higher in the suspected CS group (68.2% vs. 45.3%, p = 0.059). Moreover, there were significant differences in the medication rate of β-blockers and echocardiographic findings, including the LVEF and LVDd, between the two groups (54.5% vs. 10.7%, p < 0.001; 51.3% vs. 63.8%, p < 0.001; 49.7 mm vs. 42.7 mm, p < 0.001; respectively). However, there were no significant differences in the other clinical parameters between the two groups.

**Table 1:** Baseline characteristics.

	Suspected CS group (N = 22)	Non-suspected CS group (N = 75)	p-value
Age, years	71.0 [59.3, 79.3]	81.0 [73.0, 86.0]	0.002
Sex, male	15 (68.2%)	34 (45.3%)	0.059
Height, cm	160.3 ± 10.3	156.9 ± 9.3	0.155
Weight, kg	63.2 ± 17.9	55.1 ± 11.7	0.065
BMI, kg/m^2^	23.2 [21.2, 26.0]	22.1 [20.3, 24.6]	0.163
Hypertension	8 (36.4%)	34 (45.3%)	0.455
Dyslipidemia	9 (40.9%)	33 (44.0%)	0.797
Diabetes	5 (22.7%)	20 (26.7%)	0.710
BNP	88.1 [25.6, 275]	45.9 [29.9, 104]	0.261
Cr, mg/dL	0.84 [0.73, 1.1]	0.75 [0.66, 1.00]	0.180
eGFR	60.7 ± 22.7	61.5 ± 22.2	0.875
Paced QRS duration, ms	173.4 ± 20.1	155.8 ± 16.0	< 0.001
LVEF (%)	51.3 [37.0, 62.7]	63.8 [59.3, 69.4]	< 0.001
LAD, mm	36.0 ± 6.3	33.8 ± 5.6	0.107
LVDd, mm	49.7 ± 7.5	42.7 ± 5.0	< 0.001
β-Blocker	12 (54.5%)	8 (10.7%)	< 0.001
ACEi or ARB	6 (27.3%)	24 (32.0%)	0.673
Ca-blocker	5 (22.7%)	26 (34.7%)	0.291
Diuretics	8 (36.4%)	22 (29.3%)	0.530

Data are reported as mean ± SD, median [interquartile range], and numbers (%).

*ACEI* angiotensin-converting enzyme inhibitor, *ARB* angiotensin II receptor blocker, *BMI* body mass index, *BNP* brain natriuretic peptide, *Cr* creatinine, *CS* cardiac sarcoidosis, *eGFR* estimated glomerular filtration rate, *LAD* left atrial diameter, *LVDd* left ventricular diastolic diameter, *LVEF* left ventricular ejection fraction.

### Prevalence of patients diagnosed with CS

**Figure 2:**
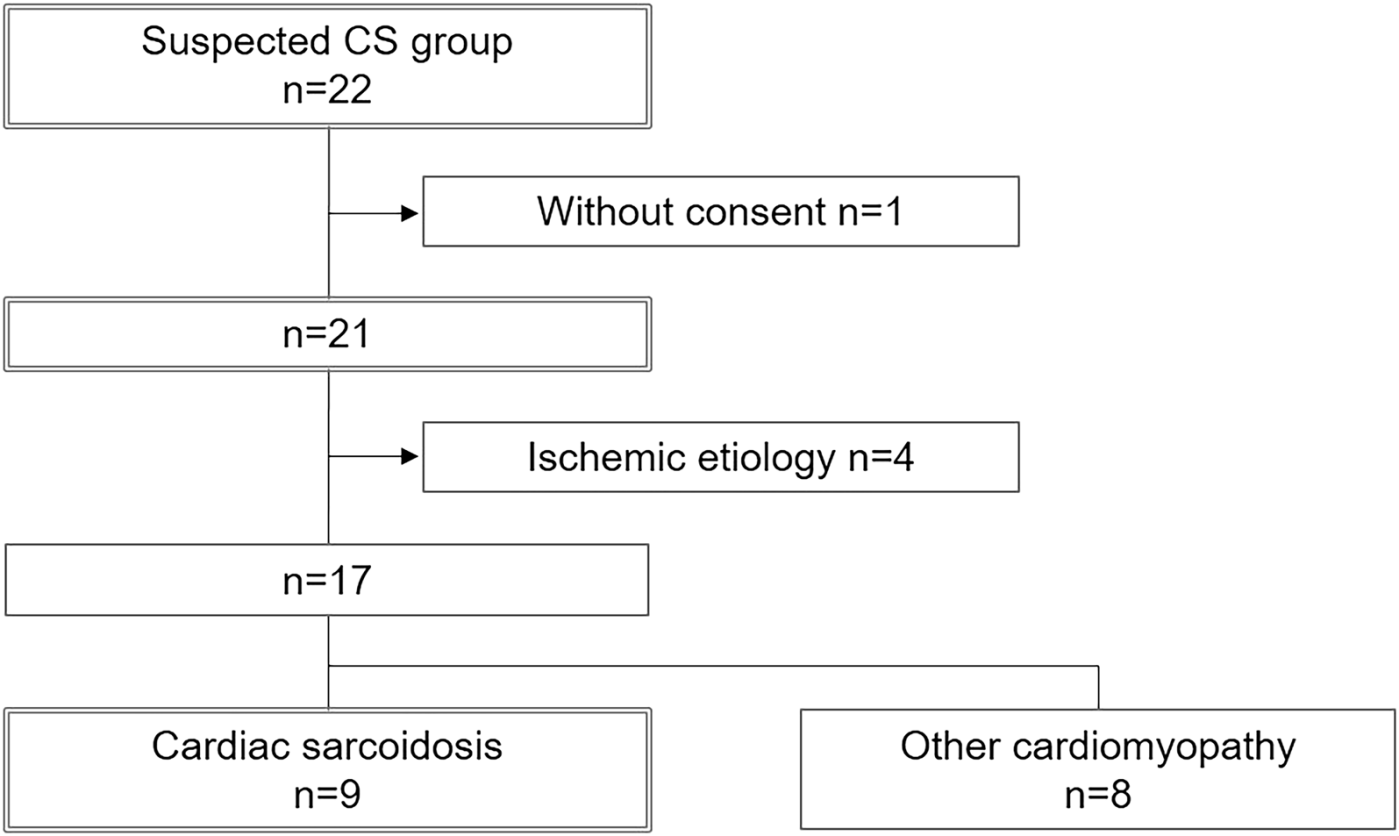
Prevalence of cardiac sarcoidosis (CS) among the patients. Among the 22 patients with suspected CS, 1 did not consent and 4 were diagnosed with ischemic cardiomyopathy by coronary CT and/or coronary angiography. The remaining 17 patients underwent cardiac 18F-FDG-PET/CT for CS diagnosis. Of these, 9 patients had a distinctive accumulation with a ‘focal’ or ‘focal on diffuse’ pattern of myocardial uptake. As a result, 9 of the 21 patients (43%) were diagnosed with CS. *CS* cardiac sarcoidosis, *CT* computed tomography, *F-FDG-PET/CT* 18F-fluoro-deoxyglucose positron-emission tomography computed tomography.

Of the 22 patients in the suspected CS group, one failed to give consent and was excluded from further examination. Of the 21 patients who provided consent, four were diagnosed with ischemic cardiomyopathy using coronary CT and/or coronary angiography. The cardiac FDG-PET/CT scans of the remaining 17 patients without ischemic cardiomyopathy were examined for the diagnosis of CS. Of these, nine patients exhibited a distinctive accumulation with a ‘focal’ or ‘focal on diffuse’ pattern of myocardial uptake. Therefore, nine of the 21 cases (43%) were diagnosed with CS (Fig. 2).

### Details of the patients diagnosed with CS

Details of the nine patients diagnosed with CS are shown in Tables 2 and 3. Table 2 presents the major and minor criteria outlined in the JCS 2016 guidelines that are indicative of cardiac involvement in sarcoidosis. The criteria consisted of (a)–(h); however, (e) gadolinium-enhanced MRI and (h) endomyocardial biopsy were not performed in any of the nine patients. All nine patients had two or more of the five major criteria, strongly suggesting the presence of cardiac involvement according to either the new or conventional guidelines. Moreover, patients 4–9 satisfied four or more of the five major criteria and the clinical diagnostic criteria for iCS.

**Table 2:** Characteristic findings suggestive of cardiac sarcoidosis.

Pt. no	Age	Sex	Major criteria							Minor criteria			Cardiac involvement	
			(a)	(b)	(c)		(d)		(e)	(f)	(g)	(h)	2006 JCS criteria	2016 JCS criteria
			AV-b	Ventricular structural abnormality	Left ventricular contractile dysfunction	LVEF	^18^F-FDG uptake	^67^Ga uptake	LGE MRI	Abnormal ECG	Myocardial SPECT	Endomyocardial biopsy		
1	59	M	+	No	Diffuse hypokinesis	44	Focal	No exam	No exam	Pacing	Detect	No exam	Yes	Yes
2	63	F	+	Left ventricular hypertrophy	No	62	Focal	No exam	No exam	Yes	No exam	No exam	Yes	Yes
3	86	M	+	Basal septal thinning	No	53	Focal on diffuse	No exam	No exam	Pacing	No exam	No exam	Yes	Yes
4	78	F	+	Basal septal thinning	Diffuse hypokinesis	34	Focal on diffuse	No exam	No exam	Pacing	No exam	No exam	Yes	Yes
5	84	M	+	Basal posterior thinning	Diffuse hypokinesis	39	Focal	No exam	No exam	Pacing	No exam	No exam	Yes	Yes
6	68	F	+	Apex ventricular aneurysm	Apex-anterior wall motion asynergy	57	Focal	No exam	No exam	Yes	No exam	No exam	Yes	Yes
7	48	M	+	Basal posterior thinning	Diffuse hypokinesis	48	Focal on diffuse	No exam	No exam	Pacing	No exam	No exam	Yes	Yes
8	79	M	+	Basal posterior thinning	Diffuse hypokinesis	49	Focal on diffuse	No exam	No exam	Pacing	Detect	No exam	Yes	Yes
9	62	M	+	Basal septal thinning	Diffuse hypokinesis	31	Focal on diffuse	Negative	No exam	Pacing	No exam	No exam	Yes	Yes

^*18*^*F-FDG* 18F-fluorodeoxyglucose, ^*67*^*Ga* Gallium-67, *AV-b* atrioventricular block, *ECG* electrocardiogram, *exam* examination, *JCS* Japanese Circulation Society, *LGE* late-gadolinium enhancement, *LVEF* left ventricular ejection fraction, *MRI* magnetic resonance imaging, *Pt. no* patient number, *SPECT* single-photon emission computed tomography.

**Table 3:** Characteristic findings suggestive of non-cardiac sarcoidosis.

Pt. no	Age	Sex	Extracardiac sarcoidosis			Characteristic laboratory findings of sarcoidosis							Diagnosis	
						(1)	(2)		(3)	(4)		(5)		
			PS	OS	Extracardiac biopsy	BHL	ACE activity	Serum lysozyme	sIL-2R level	Extracardiac ^67^Ga uptake	Extracardiac ^18^F-FDG uptake	CD4/CD8 ratio of > 3.5 in BAL fluid	2006 JCS criteria	2016 JCS criteria
1	59	M	No	No	Mediastinal lymph nodes	No	Normal	No exam	Normal	No exam	Yes	No exam	Possible	Histological (CS+, eCS+)
2	63	F	Yes	Yes	No exam	Yes	Normal	No exam	No exam	Yes	Yes	Yes	Possible	Clinical (CS+, eCS+)
3	86	M	Yes	Yes	No exam	Yes	Normal	No exam	High	No exam	Yes	Yes	Possible	Clinical (CS+, eCS+)
4	78	F	No	Yes	No exam	No	High	No exam	High	No exam	Yes	No exam	Possible	Clinical (CS+, eCS+)
5	84	M	No	No	No exam	No	Normal	No exam	Normal	No exam	No	No exam	Impossible	Isolated (CS+, eCS−)
6	68	F	No	No	No exam	No	Normal	No exam	Normal	No exam	No	No exam	Impossible	Isolated (CS+, eCS−)
7	48	M	No	No	No exam	No	Normal	No exam	Normal	No	No	No exam	Impossible	Isolated (CS+, eCS−)
8	79	M	No	No	No exam	No	Normal	Normal	High	No exam	No	No exam	Impossible	Isolated (CS+, eCS−)
9	62	M	No	No	No exam	No	Normal	No exam	No exam	No exam	No	Yes	Impossible	Isolated (CS+, eCS−)

^*18*^*F-FDG* 18F-fluorodeoxyglucose, ^*67*^*Ga* Gallium-67, *ACE* angiotensin-converting enzyme, *BAL* bronchoalveolar lavage, *BHL* bilateral hilar lymphadenopathy, *JCS* Japanese Circulation Society, *OS* ophthalmic sarcoidosis, *PS* pulmonary sarcoidosis, *Pt. No* patient number, *sIL-2R* soluble interleukin-2 receptors.

Table 3 shows the characteristic findings suggestive of sarcoidosis in other organs. Epithelioid granulomas in organs other than the heart and clinical findings suggestive of pulmonary and ophthalmic sarcoidosis were found in patients 1 and patients 2–4, respectively. In terms of the laboratory findings characteristic of sarcoidosis, bilateral hilar lymphadenopathy (BHL) was observed in two patients. Moreover, serum ACE activity was high in one patient and normal in the other eight. The serum lysozyme level was examined in one patient and was found to be normal. Additionally, serum sIL-2R levels were examined in seven patients, revealing elevated levels in three patients. Regarding extracardiac radioisotope uptake, either Gallium-67 (67 Ga) citrate scintigraphy or 18F-FDG uptake was positive in four of nine patients. Furthermore, all three patients who underwent bronchoalveolar lavage (BAL) had elevated lymphocyte rates.

### Echocardiographic and ^18^F-FDG uptake findings suggestive of CS

**Figure 3:**
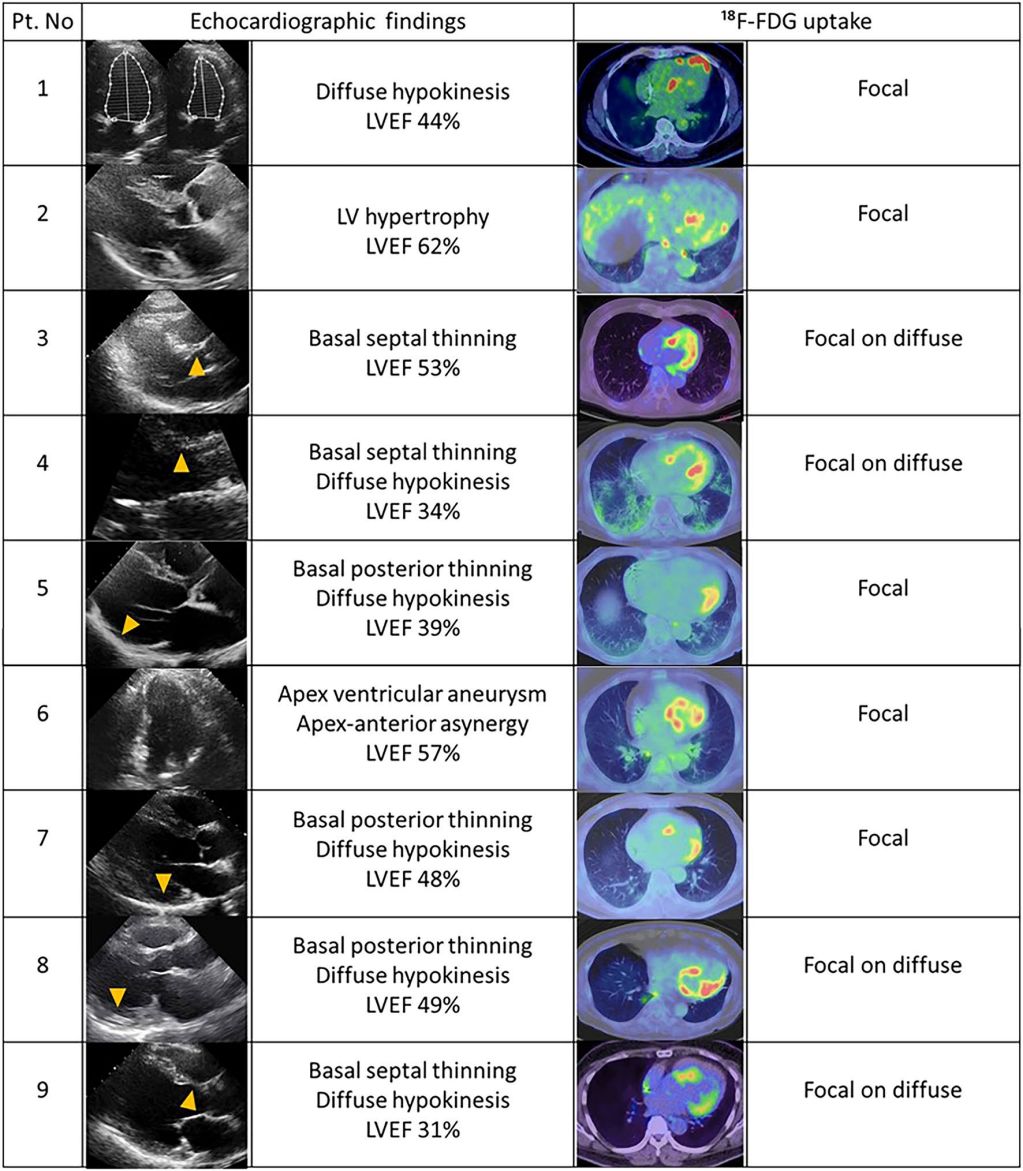
Echocardiographic and ^18^F-FDG uptake imaging suggestive of cardiac sarcoidosis. The echocardiographic findings, including ventricular structural abnormality, left ventricular contractile dysfunction, and LVEF and ^18^F-FDG imaging are noted. Patients show the following BS level prior to the FDG-PET/CT scan. Pt. No 1, BS 103 mg/dL; No 2, BS 82 mg/dL; No 3, BS 109 mg/dL; No 4, BS 60 mg/dL; No 5, BS 97 mg/dL; No 6, BS 106 mg/dL; No 7, BS 106 mg/dL; No 8, BS 89 mg/dL; No 9, BS 91 mg/dL. Yellow arrows indicate basal thinning. Pt. No corresponds to Pt. No in Table 2. *Pt. no* patient number, ^*18*^*F-FDG* 18F-fluorodeoxygluc, *LVEF* left ventricular ejection fraction, *BS* blood sugar.

The echocardiography and ^18^F-FDG uptake imaging in patients with CS are shown in Fig. 3.

### Comparison between the prevalence of CS diagnosed by the conventional guideline with the new guideline

Patient 1 was histologically diagnosed with systemic sarcoidosis with cardiac involvement. This diagnosis was established based on the presence of epithelioid granulomas in the lungs in addition to the presence of two or more of the five major criteria for cardiac involvement of sarcoidosis. Patients 2, 3, and 4 were clinically diagnosed with systemic sarcoidosis with cardiac involvement because they had clinical findings suggestive of pulmonary or ophthalmic sarcoidosis, and at least two of the following five characteristic laboratory findings of sarcoidosis: BHL, high serum ACE activity or elevated serum lysozyme levels, high serum sIL-2R levels, significant tracer accumulation on 67 Ga citrate scintigraphy or FDG-PET/CT, a high percentage of lymphocytes with a CD4/CD8 ratio of > 3.5 in BAL fluid. These findings, combined with at least two or more clinical findings, strongly suggest the above-mentioned cardiac involvement of sarcoidosis. Patients 1–4 received a definite diagnosis of CS based on both the conventional guideline and the new guidelines.

In contrast, patients 5, 6, 7, 8, and 9 were diagnosed with iCS because they satisfied the above-mentioned major criteria and all the following prerequisites: (1) No clinical findings characteristic of sarcoidosis are observed in any organs other than the heart; (2) 67 Ga scintigraphy or FDG-PET/CT reveals no abnormal tracer accumulation in any organs other than the heart; (3) A chest CT scan reveals no shadow along the lymphatic tracts in the lungs or no hilar and mediastinal lymphadenopathy; and (4) The criterion (d) and at least three other criteria of the cardiac involvement major criteria (a) to (e) are satisfied.

**Figure 4:**
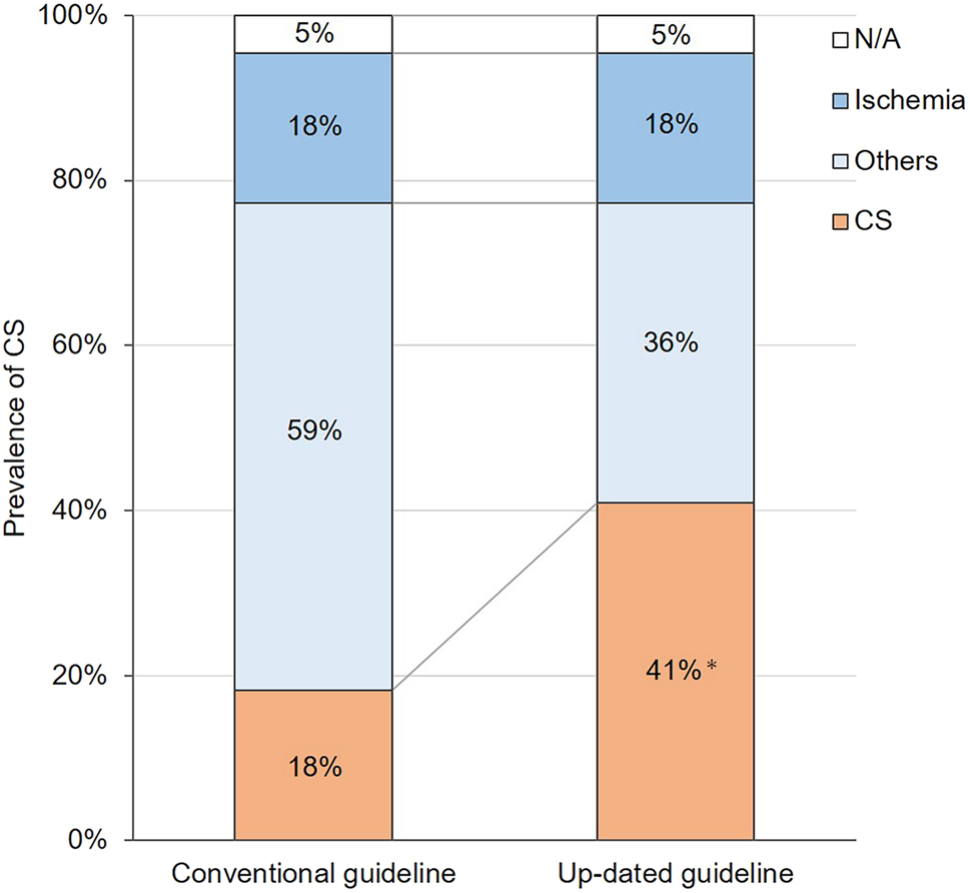
Comparison between the prevalence of CS diagnosed by the conventional guideline with the new guideline. Among the 21 patients, 9 (43%) satisfied the diagnostic criteria for CS according to the new guideline. In contrast, four patients (19%) met conventional guidelines. Approximately 2.3-fold of the patients were diagnosed with CS using the new criteria compared to the conventional criteria. *p = 0.074 with McNemar test. *CS* cardiac sarcoidosis.

As a result, five of nine cases (56%) satisfied the diagnostic criteria for iCS according to the new guideline. As shown in Fig. 4, approximately 2.3 times as many patients were diagnosed with CS using the new guideline compared with the conventional guideline.

### LVEF changes and clinical events following steroid therapy

**Figure 5:**
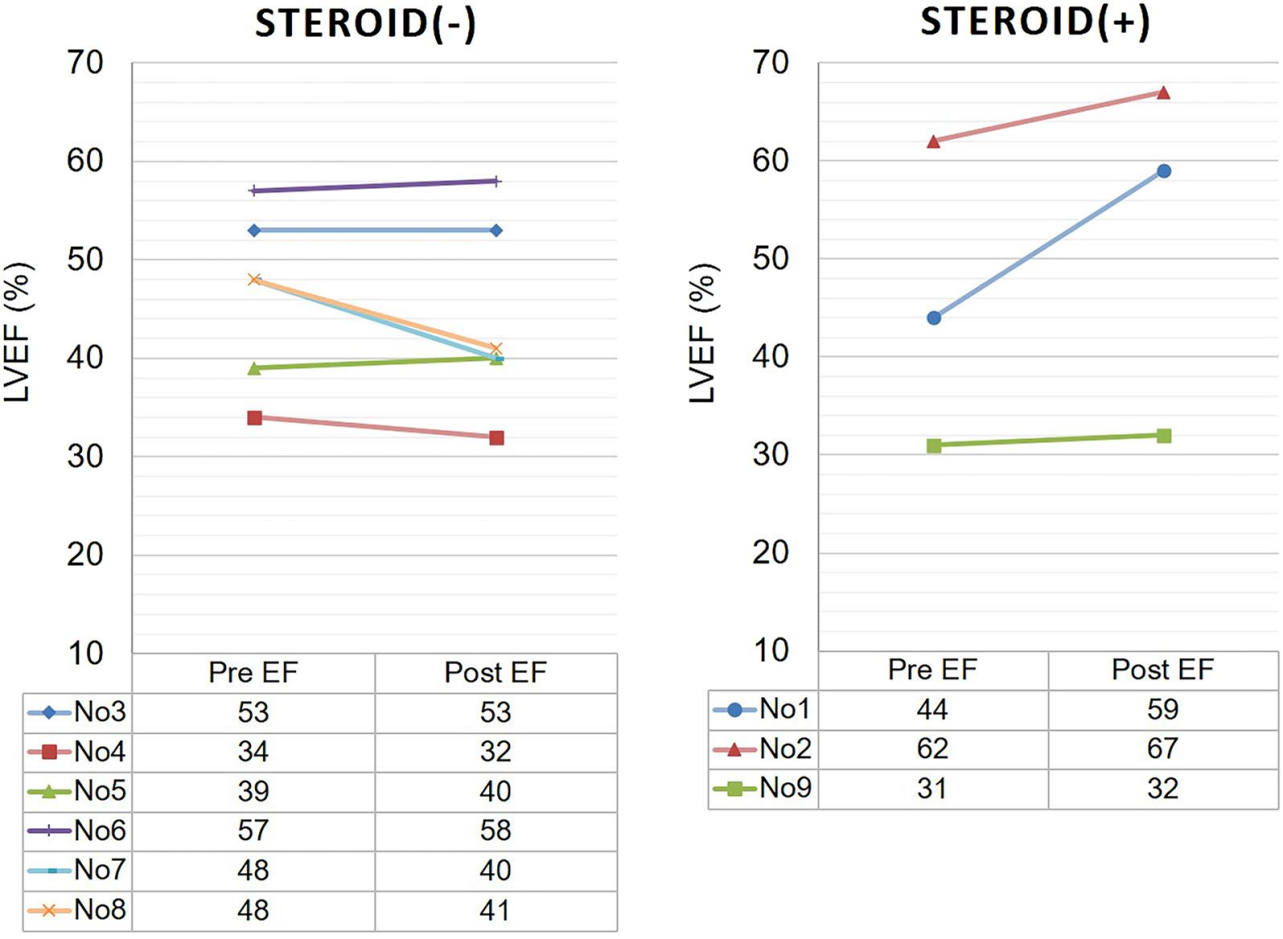
LVEF changes and clinical events following steroid therapy. In a prospective view during 36 months after CS diagnosis, three of the nine patients diagnosed with CS were treated with corticosteroids. No decrease in LVEF was observed in the patients treated with corticosteroid therapy after CS diagnosis; however, some patients who could not be treated with corticosteroid therapy had a decrease in LVEF. *CS* cardiac sarcoidosis, *EF* ejection fraction, *LVEF* left ventricular ejection fraction.

**Figure 6:**
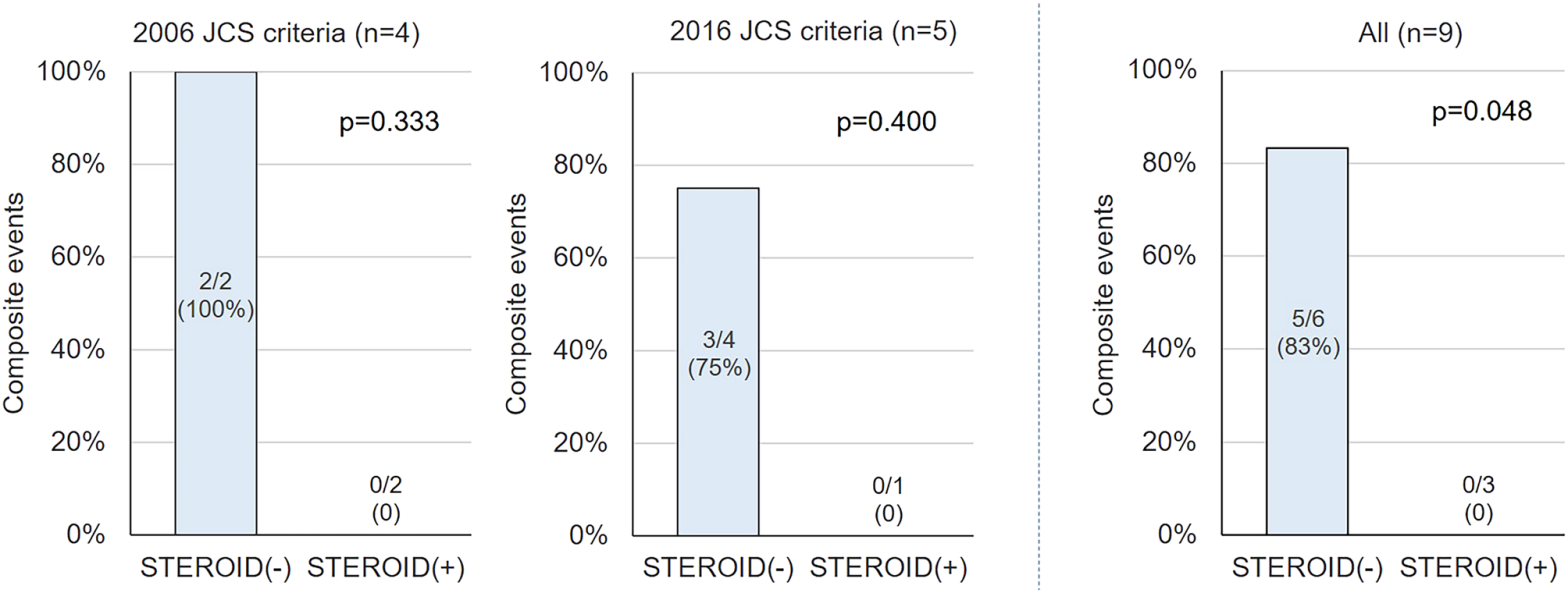
Clinical events after treatment with corticosteroid in patients with post-diagnosis CS. In patients diagnosed with CS, adverse composite outcome consists of all-cause death, heart failure hospitalization, and a substantial reduction in LVEF were not seen in the steroid treatment cohort. Conversely, in the non-steroid treatment group diagnosed with the conventional guideline, non-cardiac death (n = 1: Patient No.3) and heart failure death (n = 1: Patient No.4) were observed, and heart failure admission (n = 1: Patient No.5) and a substantial LVEF decrease (n = 2: Patient No.7, 8) were seen in the non-steroid treatment group diagnosed with the new criteria. Overall, adverse composite outcomes were significantly more common in the non-steroid treatment group than in the steroid treatment group (p = 0.048). *CS* cardiac sarcoidosis, *LVEF* left ventricular ejection fraction, *No* number.

From a prospective view over 36 months in patients with post-diagnosis CS, three of the nine patients diagnosed with CS were treated with corticosteroids. No decrease in LVEF was observed in the patients treated with corticosteroids; however, some patients who could not undergo corticosteroid therapy had a decreased LVEF (Fig. 5). Reasons for non-administration of corticosteroids are given in Supplementary Table 2. In addition, among patients diagnosed with either the conventional or new diagnostic criteria for CS, no clinical events were seen in the steroid treatment group (Fig. 6).

## Discussion

In this study, of the 97 patients implanted with a cardiac pacemaker for AV-b, 22 cases had suspected CS during a median observation period of 5.4 years after pacemaker implantation. Of these, except for one case who did not provide consent, nine of 21 cases (43%) were diagnosed with definite CS. In particular, five of these nine patients were diagnosed with iCS using FDG-PET/CT. The number of patients diagnosed with definite CS using the new guideline was approximately 2.3 times that of the conventional criteria. Moreover, three of the nine patients diagnosed with CS were treated with corticosteroids, and none of them had clinical events including a decrease in LVEF.

In the conventional guideline-based diagnosis of CS, histological evidence of granulomatous inflammation must be demonstrated in at least one organ^10,11^. Endomyocardial biopsy is currently the only technique that allows the pathological diagnosis of CS; nevertheless, its diagnostic performance is limited, at almost 20%^14^. Especially in patients with a cardiac pacemaker, it is sometimes difficult to perform endomyocardial biopsies because the pacing lead can obstruct the retrieval of endomyocardial tissues. Therefore, definite CS is difficult to diagnose in some patients, even when clinical findings are strongly suggestive of CS^15,16^.

The new guideline has several potential advantages for the early diagnosis of CS compared to the conventional criteria. First, the new guidelines identified abnormal accumulation of FDG-PET/CT in the myocardium as a major criterion, as well as late-gadolinium enhancement of the myocardium in gadolinium-enhanced MRI, based on recent advancements in imaging techniques and accumulated clinical experience^9,17–20^. FDG-PET/CT and MRI are useful modalities for the evaluation of inflammatory lesions in patients with suspected CS^9,17–20^, which help us diagnose CS without histological findings. Second, the definition of iCS was established for the first time^8^. Until now, cases with findings strongly suggestive of clinical CS might not have been diagnosed with CS before disease progression because they did not meet the criteria of CS^21^. According to the new definition, iCS can be diagnosed without histology if the four above-mentioned items are met^8^.

In this study, the new guideline resulted in almost 2.3 times as many cases being diagnosed with CS compared to the conventional criteria. Nine patients were diagnosed with CS according to the new guideline, including five newly diagnosed iCS cases using FDG-PET/CT, while only four were diagnosed with CS according to the conventional criteria. MRI is also helpful in diagnosing CS^9,17–20^; however, it is challenging to perform in patients with cardiac pacemaker implantation. FDG-PET/CT overcomes this limitation as it is safe to perform in patients with cardiac pacemaker implantation^9^, and all patients with iCS in this study were diagnosed using FDG-PET/CT. The new guideline, especially the use of FDG-PET/CT, could potentially offer a definitive diagnosis of CS in the absence of histological evidence. This approach might aid in achieving an earlier definitive diagnosis of CS and could have a positive impact on patient outcomes.

The present study had some limitations. First, this was a retrospective observational study involving a small number of patients from a single center. Second, the diagnostic approach discussed in the present study may not accurately present real-world clinical settings, as it deviates from international guidelines which advocate for the assessment of CS in patients exhibiting signs of extra-cardiac sarcoidosis. Moreover, by including only patients with echocardiographic findings such as anatomical or functional cardiac abnormalities, the study potentially underestimated the prevalence of CS. Third, the cardiac dysfunction group may have included patients with undiagnosed CS due to false-negative PET results. Additionally, the excluded cases diagnosed with ischemic heart disease or the ‘diffuse’ pattern accumulation might have had concurrent CS^22^. This might lead to a low estimation of the actual incidence of CS. In such cases, as well as in situations where cardiac magnetic resonance is unavailable, for a more accurate diagnosis of CS, rest perfusion imaging should have been included. Finally, we acknowledge the concern that the new guideline may lead to an increased incidence of false positives. The accurate interpretation of PET imaging results is challenging, especially in facilities without specialists in CS. Despite these challenges, we believe that the new guidelines will improve the detection of CS. Therefore, we wish to emphasize that inadequate examination and suboptimal preparation before a PET/CT scan must be prevented to avoid resulting in a higher rate of false-positive diagnoses of CS. Larger prospective studies are required to gain further insights.

In conclusion, patients with CS were latent among those with cardiac pacemakers for AV-b. The new guideline using FDG-PET/CT enabled the definitive diagnosis of CS, including iCS, leading to approximately 2.3 times the number of diagnoses of CS when compared to the conventional criteria. These findings may therefore contribute to the early diagnosis of CS and improve patient clinical conditions.

## Methods

### Study population and flow

This was a retrospective observational study. We examined all patients implanted with a cardiac pacemaker due to AV-b who were attending the outpatient pacemaker clinic at the Aichi Medical University Hospital. Those with implantable cardioverter-defibrillators or cardiac resynchronization therapy devices were excluded. The patients underwent periodical follow-up echocardiography between June 2018 and May 2019 and were divided into two groups according to the presence or absence of echocardiographic findings suggestive of CS, based on the JCS 2016 Guideline on the Diagnosis and Treatment of CS^8^. The suspected CS group included patients with AV-b with abnormal echocardiographic findings, including a left ventricular ejection fraction (LVEF) < 50% and/or structural abnormalities such as local wall motion asynergy and/or local ventricular aneurysm. In contrast, the non-suspected CS group included patients with AV-b with a LVEF ≥ 50% and lacked structural abnormalities. The echocardiographic findings were independently assessed by two cardiologists (S.T. and Y.N.) and echocardiographic technicians. The prevalence of CS was then confirmed by close examination, and the LVEF change and clinical events were observed following standard steroid therapy.

This study was conducted in accordance with the Declaration of Helsinki. The study protocol was approved by the Ethics Committee of Aichi Medical University Hospital (approved ID: 2021-508). Informed consent to use patient data was obtained from all participants prior to study through an opt-out mechanism. Those who did not explicitly opt-out were considered to have given consent.

### Clinical data acquisition

Demographic and clinical data of the patients, as well as blood sample data, were the latest available information at the time of group assignment. Cut-off values of brain natriuretic peptide, creatinine, angiotensin-converting enzyme (ACE), serum lysozyme, and soluble interleukin-2 receptors (sIL-2Rs) were decided as 18.4 pg/mL, 1.04 mg/mL (Female: 0.79 mg/mL), 21.4 U/L, 10.2 µg/mL, and 500 U/mL, respectively. Moreover, echocardiographic technicians measured the presence of any ventricular septal thinning (< 4 mm thick at 10 mm from the aortic annulus in the left ventricular long-axis view), ventricular aneurysm, and local wall motion abnormality, as well as the values of left ventricular end-diastolic diameter, LVEF, and left atrial diameter^22^. Additionally, the presence or absence of systemic involvement was determined by respiratory, ophthalmologic, dermatologic, and other examinations of the patients. For patients with suspected CS who required a differential diagnosis, coronary CT angiography, invasive coronary angiography, or perfusion scintigraphy were added. Patients with no abnormalities in these tests underwent FDG-PET/CT scanning for the diagnosis of CS as part of routine medical practice.

### FDG-PET/CT procedure and image analysis

Before undergoing the FDG-PET/CT scan, patients were instructed to adhere to a high-fat, low-carbohydrate diet for 48 h before the examination, followed by an 18-h fasting period immediately before the examination. Additionally, blood glucose levels were confirmed to be below 150 mg/dL prior to the FDG-PET/CT scan. Patients received low doses (50 IU/kg) of unfractionated heparin before the intravenous administration of ^18^F-FDG. FDG-PET/CT imaging commenced after a 90-min uptake period^23^, and the PET/CT data were analyzed by two cardiologists (S.T. and N.Y.) and radiologists. Disease involvement sites were defined as positive if abnormal FDG uptake occurred in a pattern consistent with sarcoidosis. During the visual assessment of cardiac FDG-PET/CT images, the presence of ‘focal’ or ‘focal on diffuse’ myocardial uptake patterns were defined as positive, while ‘diffuse’ or ‘none’ patterns were indicative of physiological uptake and were considered negative^17^.

### Prevalence of patients diagnosed with CS

The prevalence of patients diagnosed with CS was compared between the conventional and new guidelines.

### LVEF changes and clinical events following steroid therapy

After the definitive diagnosis of CS by PET/CT, changes in LVEF and clinical events occurring within the first 36 months were observed in patients who consented to steroid therapy as part of routine clinical care. Clinical events were evaluated by the composite outcome of all-cause death, hospitalization due to heart failure, and a substantial reduction in LVEF.

### Statistical analysis

Continuous variables were expressed as the mean ± standard deviation (SD) for normally distributed variables or the median (interquartile range) for non-parametric variables. The normality of the data was assessed using the Shapiro–Wilk test. Comparisons of normally distributed variables were performed using the independent samples unpaired t-test. Non-parametric variables were analyzed using the Mann–Whitney U-test. Categorical data were presented as numbers (%) and were analyzed using the chi-square test. Fisher’s exact test was used to compare categorical variables, with expected values less than 5. The ratio of diagnoses of CS based on conventional to new diagnostic criteria was analyzed using the McNemar test. All p-values were two-tailed, with p-values < 0.05 considered as statistically significant. All statistical analyses were performed using IBM SPSS Statistics for Windows version 25 (IBM Corp., Armonk, NY, USA).

### Supplementary Information

Supplementary Tables.

## Data Availability

The data that support the findings of this study are not openly available due to reasons of sensitivity and are available from the corresponding author upon reasonable request.
