# Supplementary material for: Utility of new FDG-PET/CT guidelines for diagnosing cardiac sarcoidosis in patients with implanted cardiac pacemakers for atrioventricular block
Source: Sci Rep. 2024 Apr 3;14:7825. doi: 10.1038/s41598-024-58475-z (PMC10991404; doi:10.1038/s41598-024-58475-z)
Supplement: Supplementary file 1 — Supplementary Tables. [file 41598_2024_58475_MOESM1_ESM.pptx]

## Slide 1
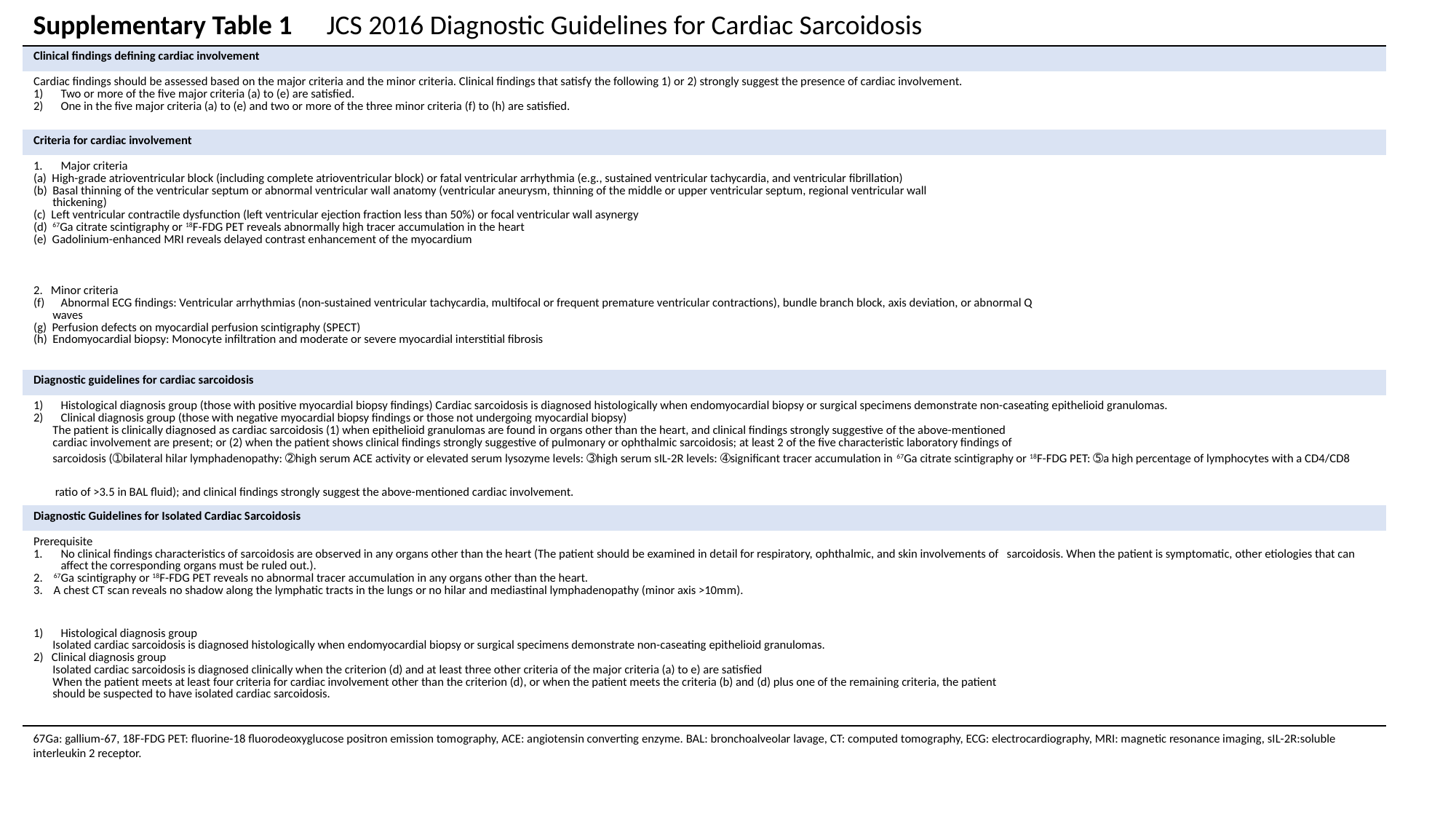

Supplementary Table 1
JCS 2016 Diagnostic Guidelines for Cardiac Sarcoidosis
| Clinical findings defining cardiac involvement |
| --- |
| Cardiac findings should be assessed based on the major criteria and the minor criteria. Clinical findings that satisfy the following 1) or 2) strongly suggest the presence of cardiac involvement. Two or more of the five major criteria (a) to (e) are satisfied. One in the five major criteria (a) to (e) and two or more of the three minor criteria (f) to (h) are satisfied. |
| Criteria for cardiac involvement |
| Major criteria (a) High-grade atrioventricular block (including complete atrioventricular block) or fatal ventricular arrhythmia (e.g., sustained ventricular tachycardia, and ventricular fibrillation) (b) Basal thinning of the ventricular septum or abnormal ventricular wall anatomy (ventricular aneurysm, thinning of the middle or upper ventricular septum, regional ventricular wall thickening) (c) Left ventricular contractile dysfunction (left ventricular ejection fraction less than 50%) or focal ventricular wall asynergy (d) 67Ga citrate scintigraphy or 18F-FDG PET reveals abnormally high tracer accumulation in the heart (e) Gadolinium-enhanced MRI reveals delayed contrast enhancement of the myocardium |
| 2. Minor criteria Abnormal ECG findings: Ventricular arrhythmias (non-sustained ventricular tachycardia, multifocal or frequent premature ventricular contractions), bundle branch block, axis deviation, or abnormal Q waves (g) Perfusion defects on myocardial perfusion scintigraphy (SPECT) (h) Endomyocardial biopsy: Monocyte infiltration and moderate or severe myocardial interstitial fibrosis |
| Diagnostic guidelines for cardiac sarcoidosis |
| Histological diagnosis group (those with positive myocardial biopsy findings) Cardiac sarcoidosis is diagnosed histologically when endomyocardial biopsy or surgical specimens demonstrate non-caseating epithelioid granulomas. Clinical diagnosis group (those with negative myocardial biopsy findings or those not undergoing myocardial biopsy) The patient is clinically diagnosed as cardiac sarcoidosis (1) when epithelioid granulomas are found in organs other than the heart, and clinical findings strongly suggestive of the above-mentioned cardiac involvement are present; or (2) when the patient shows clinical findings strongly suggestive of pulmonary or ophthalmic sarcoidosis; at least 2 of the five characteristic laboratory findings of sarcoidosis (➀bilateral hilar lymphadenopathy: ➁high serum ACE activity or elevated serum lysozyme levels: ➂high serum sIL-2R levels: ➃significant tracer accumulation in 67Ga citrate scintigraphy or 18F-FDG PET: ➄a high percentage of lymphocytes with a CD4/CD8 　　 　 ratio of >3.5 in BAL fluid); and clinical findings strongly suggest the above-mentioned cardiac involvement. |
| Diagnostic Guidelines for Isolated Cardiac Sarcoidosis |
| Prerequisite No clinical findings characteristics of sarcoidosis are observed in any organs other than the heart (The patient should be examined in detail for respiratory, ophthalmic, and skin involvements of sarcoidosis. When the patient is symptomatic, other etiologies that can affect the corresponding organs must be ruled out.). 2. 67Ga scintigraphy or 18F-FDG PET reveals no abnormal tracer accumulation in any organs other than the heart. 3. A chest CT scan reveals no shadow along the lymphatic tracts in the lungs or no hilar and mediastinal lymphadenopathy (minor axis >10mm). |
| Histological diagnosis group Isolated cardiac sarcoidosis is diagnosed histologically when endomyocardial biopsy or surgical specimens demonstrate non-caseating epithelioid granulomas. 2) Clinical diagnosis group Isolated cardiac sarcoidosis is diagnosed clinically when the criterion (d) and at least three other criteria of the major criteria (a) to e) are satisfied When the patient meets at least four criteria for cardiac involvement other than the criterion (d), or when the patient meets the criteria (b) and (d) plus one of the remaining criteria, the patient should be suspected to have isolated cardiac sarcoidosis. |
67Ga: gallium-67, 18F-FDG PET: fluorine-18 fluorodeoxyglucose positron emission tomography, ACE: angiotensin converting enzyme. BAL: bronchoalveolar lavage, CT: computed tomography, ECG: electrocardiography, MRI: magnetic resonance imaging, sIL-2R:soluble interleukin 2 receptor.

## Slide 2
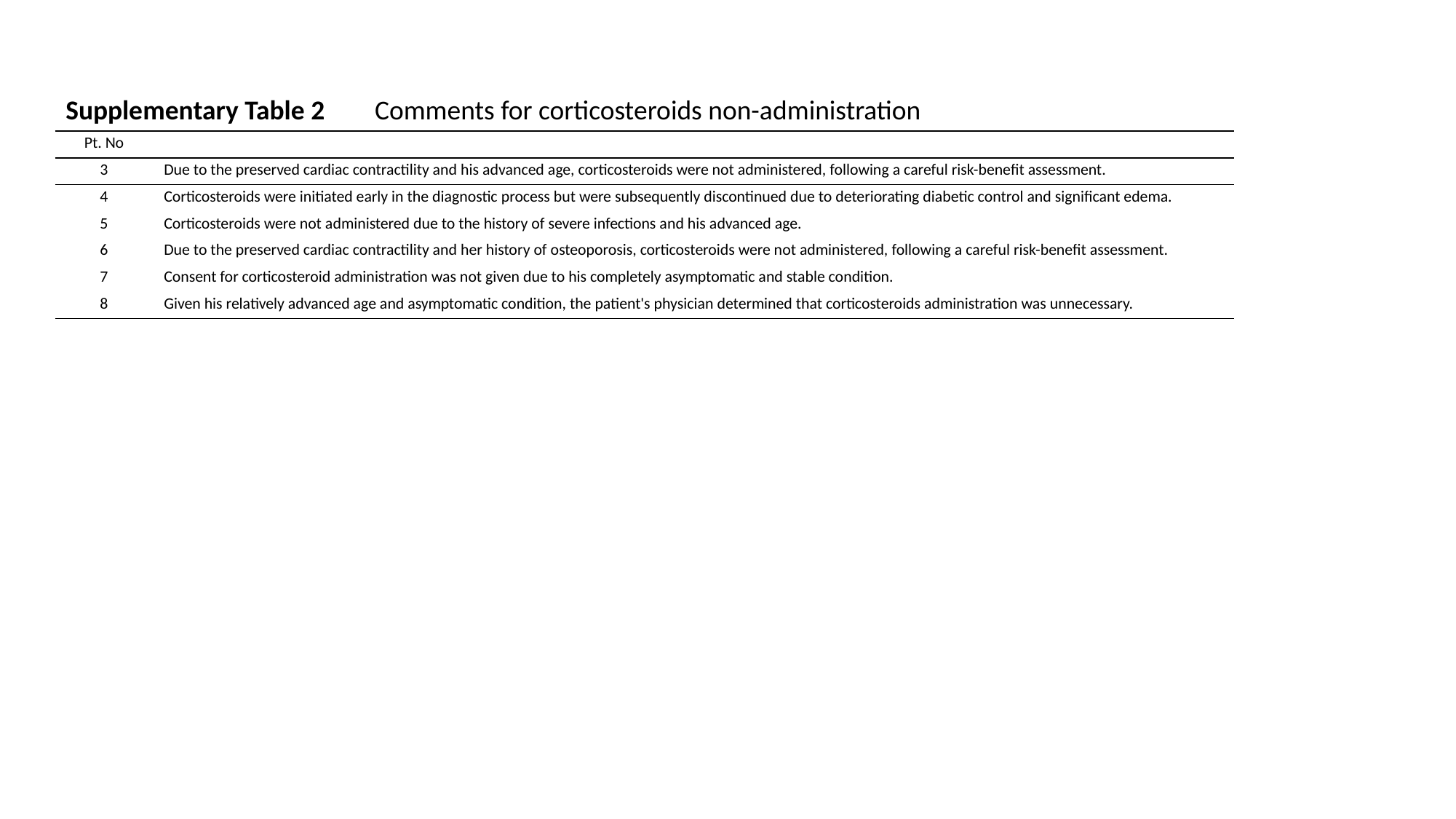

Supplementary Table 2
Comments for corticosteroids non-administration
| Pt. No | |
| --- | --- |
| 3 | Due to the preserved cardiac contractility and his advanced age, corticosteroids were not administered, following a careful risk-benefit assessment. |
| 4 | Corticosteroids were initiated early in the diagnostic process but were subsequently discontinued due to deteriorating diabetic control and significant edema. |
| 5 | Corticosteroids were not administered due to the history of severe infections and his advanced age. |
| 6 | Due to the preserved cardiac contractility and her history of osteoporosis, corticosteroids were not administered, following a careful risk-benefit assessment. |
| 7 | Consent for corticosteroid administration was not given due to his completely asymptomatic and stable condition. |
| 8 | Given his relatively advanced age and asymptomatic condition, the patient's physician determined that corticosteroids administration was unnecessary. |
